# Fluorescence lifetime imaging ophthalmoscopy (FLIO) of patients with neovascular age-related macular degeneration before and after treatment with intravitreal ranibizumab

**DOI:** 10.1007/s00417-025-07103-1

**Published:** 2026-01-20

**Authors:** Svenja Rebecca Sonntag, Stella Fries, Stefanie Gniesmer, Salvatore Grisanti, Yoko Miura

**Affiliations:** 1 Department of Ophthalmology, University of Lübeck, Ratzeburger Allee 160, 23538 Lübeck, Germany; 2 Institute of Biomedical Optics, University of Lübeck, Lübeck, Germany; 3 Medical Laser Center Lübeck, Lübeck, Germany

**Keywords:** Age-related macular degeneration, FLIO, Anti-VEGF, Ranibizumab, Metabolism

## Abstract

**Purpose:**

Neovascular age-related macular degeneration (AMD) is a leading cause of severe vision loss worldwide. Although intravitreal anti-vascular endothelial growth factor (anti-VEGF) injections improve outcomes, frequent treatments are burdensome and responses vary. This study investigates changes in macular fluorescence lifetime before and after anti-VEGF therapy using fluorescence lifetime imaging ophthalmoscopy (FLIO), and explores FLIO parameters as potential biomarkers for treatment response.

**Methods:**

Twenty patients with neovascular AMD underwent FLIO imaging (excitation: 473 nm; emission: short spectral channel [SSC]: 498–560 nm; long spectral channel [LSC]: 560–720 nm) and macular OCT before and 4–6 weeks after ranibizumab injection. Fluorescence lifetime components (mean τ_m_, short τ_1_, long τ_2_), retinal thickness (RT), and best-corrected visual acuity (BCVA, logMAR) were compared. Analysis focused on central (C), inner ring (IR), and outer ring (OR) regions of the ETDRS grid. Spearman correlation was used to assess relationships between parameter changes.

**Results:**

Changes in FLIO parameters post-treatment varied individually without a consistent trend. However, statistically significant correlations were found between changes in BCVA (∆logMAR) and changes in τ_1_ in the central area (*p* = 0.030) as well as τ_2_ in the inner ring (*p* = 0.040) in the SSC, with greater BCVA improvement associated with shorter fluorescence lifetimes. No significant correlation was observed between RT and BCVA.

**Conclusion:**

FLIO parameters correlated with visual acuity changes, while retinal thickness did not. This suggests that FLIO may capture treatment-induced alterations beyond structural changes, potentially reflecting metabolic processes. FLIO may therefore serve as a valuable adjunct tool for monitoring anti-VEGF therapy response in neovascular AMD.

## Key messages

**What is known:**

- More than 90% of AMD cases with severe vision loss are attributed to neovascular age-related macular degeneration (nAMD)

- Intravitreal Injections (IVI) are used for treatment, but due to unknown reasons therapeutic response is heterogeneous and a subset of patients exhibits suboptimal or absent anatomical and functional improvement

**What is new:**

- Our study analyzes for the first time the treatment effect of IVIs with fluorescence lifetime imaging ophthalmoscopy (FLIO)

- The study shows that change in fluorescence lifetimes after IVI correlates with improvement of vision, whereas no correlation was found between visual acuity and retinal thickness

- FLIO could therefore be a useful tool to support OCT in monitoring anti-VEGF therapy in neovascular AMD

## Introduction

Age-related macular degeneration (AMD) is a leading cause of severe vision loss in western industrialized countries. While approximately 85–90% of AMD cases are of the dry form [1, 2], the prevalence of neovascular AMD (nAMD) remains substantial, affecting around 1.75 million people in the USA alone [3]. The disease course in nAMD is typically rapid and often results in significant vision loss [3]. In fact, over 90% of severe vision loss cases in AMD are attributable to nAMD [4].

nAMD is characterized by the migration of endothelial cells from the choriocapillaris into the neuroretina. This leads to the formation of macular neovascularization (MNV), which is often accompanied by intra- or subretinal fluid, hemorrhage, and eventually retinal scarring [5].

Currently, vascular endothelial growth factor (VEGF) inhibitors are a cornerstone in the treatment of nAMD, significantly improving patient outcomes [6, 7]. These agents neutralize tissue VEGF, promote regression of MNV and reduce vascular permeability [8, 9]. Consequently, they decrease exudative changes, enhancing the retinal microenvironment and potentially improving retinal cell function. Despite their effectiveness in targeting the central pathogenic mechanism of nAMD, VEGF also plays an essential role in maintaining normal vasculature and cellular homeostasis [10, 11]. Accordingly, the long-term effects of sustained VEGF blockade on chorioretinal tissues have been examined in both experimental and clinical studies [12–14]. Laboratory evidence suggests that anti-VEGF molecules can be transported within retinal pigment epithelium (RPE) cells, potentially affecting RPE function and contributing to outer retinal and choroidal atrophy over time [15].

Clinically, therapeutic responses to anti-VEGF treatment vary widely. Some patients show suboptimal or no anatomical and functional improvement, and the factors influencing this variability are not fully understood [16]. Given the psychological burden of frequent intravitreal injections (IVI) and the high associated healthcare costs, there is an urgent need for more sensitive and early indicators of retinal response to therapy [17, 18]. Current monitoring primarily relies on OCT-based morphological endpoints, which may miss subtle metabolic or functional retinal changes. Therefore, integrating biomarkers that reflect retinal metabolism and function could be crucial to optimizing treatment strategies and addressing these challenges.

Fluorescence lifetime imaging ophthalmoscopy (FLIO) is a non-invasive diagnostic technique that measures the fluorescence lifetime (FLT) of intrinsic retinal fluorophores using ultrashort 473 nm laser pulses [19, 20]. FLIO may measure the FLT of natural fluorophores in the fundus, including bisretinoids, collagen, elastin, macular pigments, blood cells, melanin, and metabolic coenzymes such as flavin adenine dinucleotide (FAD) [19–23]. This unique capability allows FLIO to capture not only structural but also metabolic changes in the retina [24–26].

Previous FLIO studies in AMD have demonstrated alterations in FLT associated with various AMD-related pathologies such as drusen [27], subretinal drusenoid deposites [28, 29], pigment epithelial detachment [30], and geographic atrophy [31–33]. Early FLIO biomarkers of AMD have also been identified, including correlations with delayed rod-mediated dark adaptation [34]. However, no studies have investigated FLT changes in response to pharmacological treatment in AMD.

The aim of this study was to evaluate whether anti-VEGF therapy induces changes in macular FLT in patients with nAMD, using FLIO to explore its potential as a tool for monitoring metabolic treatment response. Specifically, we assessed FLT in patients receiving ranibizumab treatment before and after therapy.

## Methods

### Study design and participants

This monocentric, single-arm, prospective, longitudinal observational study was conducted at the Department of Ophthalmology, University Hospital Schleswig-Holstein, Campus Lübeck. Between January 2021 and January 2022, 20 participants with neovascular AMD (nAMD) were enrolled. Patients ranged in age from 72 to 93 years (mean 82 ± 4.7 years), including 12 females and 8 males. All participants were scheduled for intravitreal ranibizumab (Lucentis) therapy. Inclusion criteria for study participation were the ability to consent, nAMD with ranibizumab (Lucentis) treatment. Exclusion criteria were subjects with other retinal diseases, relevant media opacity, and a narrow chamber angle that would not allow the drug-induced mydriasis. It is notable, that non-inclusion was primarily related to the use of alternative anti-VEGF agents or lack of consent.

The study was positively reviewed by the ethics committee of the University of Lübeck and conducted in accordance with the ethical standards stated in the Declaration of Helsinki.

## Procedure

After obtaining written informed consent, intraocular pressure was measured using non-contact air tonometry, followed by refractometry and assessment of best-corrected visual acuity (BCVA) as part of the routine pre-injection evaluation. In the absence of any contraindications, such as narrow anterior chamber angles, pharmacologic pupil dilation was performed bilaterally in accordance with the standard protocol. BCVA measurements were recorded in logMAR and used for subsequent analysis.

Before treatment, all patients underwent fundus ophthalmoscopy, optical coherence tomography (OCT; Spectralis OCT, Heidelberg Engineering, Heidelberg), and fluorescence lifetime imaging ophthalmoscopy (FLIO) of the macula. Following these assessments, an intravitreal injection of ranibizumab (Lucentis, Novartis Pharma, Basel; 0.5 mg/0.05 ml) was administered. The same imaging procedures were repeated 4 to 6 weeks after treatment.

## Fluorescence lifetime imaging ophthalmoscopy (FLIO)

FLIO (Heidelberg Engineering, Heidelberg) employs a scanning, picosecond-pulsed laser (70 ps pulse duration, 473 nm wavelength) operating at a repetition rate of 80 MHz to excite intrinsic retinal fluorophores. Fluorescence lifetime (FLT) measurements are based on the time-correlated single photon counting (TCSPC) technique.

Emission photons are detected by two highly sensitive hybrid detectors (HPM-100-40; Becker & Hickl, Berlin) across two spectral channels: a short spectral channel (SSC: 498–560 nm) and a long spectral channel (LSC: 560–720 nm). These detectors are connected to a TCSPC module (SPC-150, Becker & Hickl) for precise photon counting. An integrated infrared laser-based eye tracking system compensates for eye movements during image acquisition.

Measurements were performed in a darkened room. Imaging continued until a photon count of 1,000 photons per pixel was reached in the foveal center for both spectral channels, ensuring adequate signal quality for reliable FLT analysis.

### OCT data analysis

Retinal thickness (RT), measured in micrometers (µm), was obtained from macular OCT scans. Automated segmentation provided by the OCT software was reviewed and manually corrected when necessary to ensure accuracy. For regional analysis, the Early Treatment Diabetic Retinopathy Study (ETDRS) grid was applied, centered on the fovea, and divided into three concentric zones: a central area (C, 1 mm diameter), an inner ring (IR, 3 mm diameter) and an outer ring (OR, 6 mm diameter).

The average RT within each zone, as calculated by the OCT software, was used for subsequent statistical analysis.

### FLIO data analysis

Photon count data was processed using SPCImage software (version 8.0 NG, Becker and Hickl). A detailed description of the software and its algorithms has been published previously [35]. At each pixel (256 × 256 resolution), the fluorescence decay curve was fitted using a biexponential decay model with a binning factor of 1. The resulting decay curve is described as the sum of two exponential components:

$$\:f\left(t\right)=\sum\:_{n=1}^{2}{\alpha\:}_{i}\cdot\:{e}^{-t/{\tau\:}_{i}}$$

where τ_1_ and τ_2_ represent the FLTs of the short and long exponential components, respectively, and α_1_ and α_2_ are their corresponding amplitudes. SPCImage also calculates the amplitude-weighted mean FLT τ_m_ as followes:

$$\:{\tau\:}_{m}=\frac{{\alpha\:}_{1}\cdot\:{\tau\:}_{1}+{\alpha\:}_{2}\cdot\:{\tau\:}_{2}}{{\alpha\:}_{1}+{\alpha\:}_{2}}$$

The obtained values of the FLT parameters (τ_1_, τ_2_, τ_m_) for each pixel position and the intensity image from both channels were exported for further analysis in the software FLIO-Reader (ARTORG Center for Biomedical Engineering Research, University of Bern, Switzerland).

**Fig. 1:**
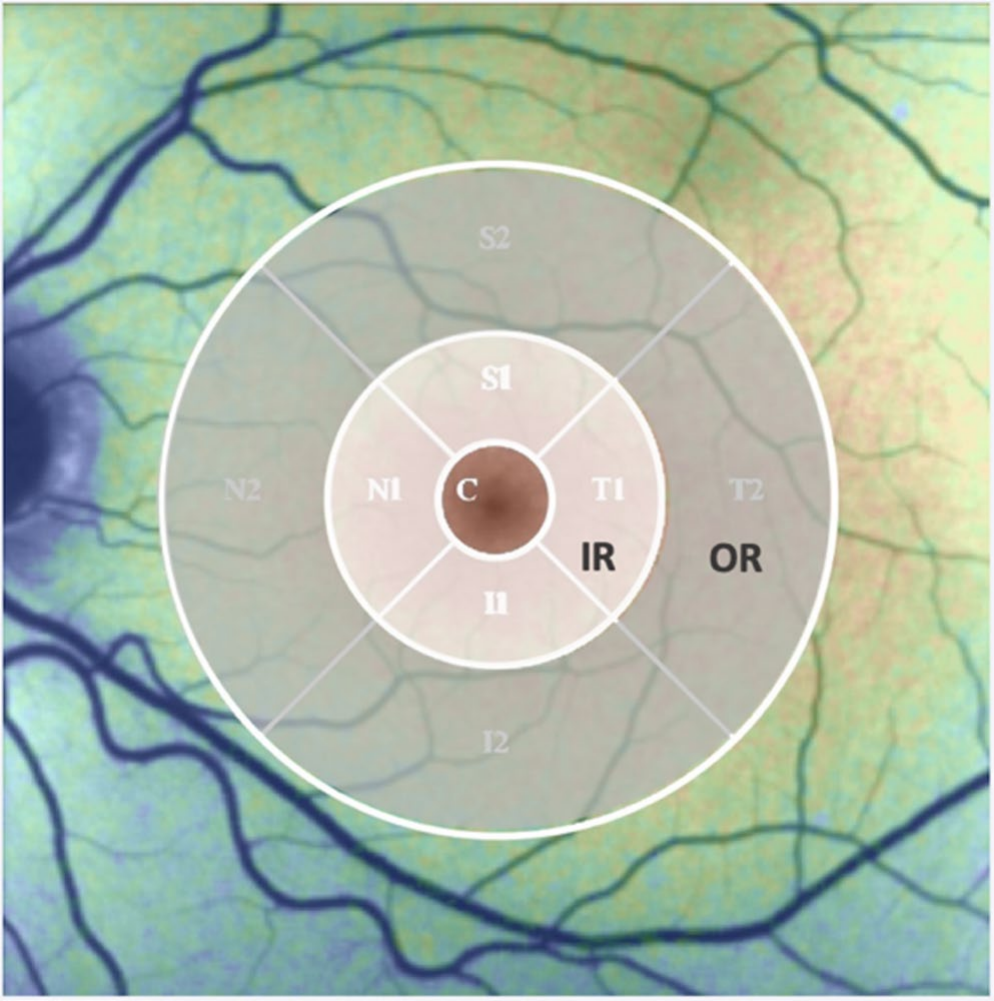
ETDRS grid in the FLIO-reader with the fovea (**C**), the inner ring (IR) and the outer ring (OR) [24]

FLIO-reader applies the ETDRS grid for regional analysis. The mean and standard deviation of τ_1_, τ_2_ and τ_m_ were calculated for each region (C, IR, OR, Fig. 1).

### Statistical analysis

The above data were collected in Microsoft^®^ Excel and imported into the statistical software Jamovi (version 1.6.15.0). Means and standard deviations were calculated for each time point and both spectral channels for the parameters τ_1_, τ_2_, τ_m_, BCVA and RT. Differences between time points (Δ τ_1_, Δ τ_2_ , Δ τ_m_ , ΔBCVA, and ΔRT) were then assessed.

The normality of the data was proved by the Shapiro-Wilk test. Depending on the distribution, differences of τ_1_, τ_2_, τ_m_, BCVA and RT between both time point were analyzed separately for the central area, IR and OR using either the Student’s t-test (for normally distributed data) or the Mann-Whitney U test (for non-normally distributed data).

In addition, Spearman correlation analysis was conducted to examine the correlations between the changes (Δ) in the above parameters across the ETDRS subfields, with Spearman’s correlation coefficient (ρ) calculated for each comparison. A p-value of < 0.05 was considered statistically significant.

## Results

### Characteristic of study cohort

As described above, the study group consisted of 20 subjects with nAMD, including 8 men (40%) and 12 women (60%). The average age was 82 ± 4.7 years.

All patients were scheduled to receive three consecutive monthly intravitreal ranibizumab injections due to the neovascular activity assessed with OCT. In 52.6% of the cases, FLIO and OCT was performed before and after the first injection, in 31.6% around the second injection and in 15.8% around the third injection. The average time interval between the two measurements was 29.8 ± 3.4 days. Overall, the majority of the patient population had been diagnosed with nAMD for several years, with the mean disease duration of 3.1 years.

### FLT (τ_1_, τ_2_, τ_m_) before and after IVI

A comparison analysis revealed no significant differences in τ_1_, τ_2_, or τ_m_ between pre- and post-IVI measurements in both spectral channels and in all three areas (Table 1).

**Table 1.**
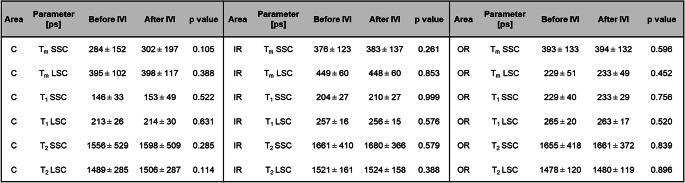
Changes in FLIO lifetime parameters (τ₁, τ₂, τ ) before and after intravitreal ranibizumab IVI across retinal regions in both spectral channels

### Retinal thickness (RT) and BCVA before and after IVI

**Fig. 2:**
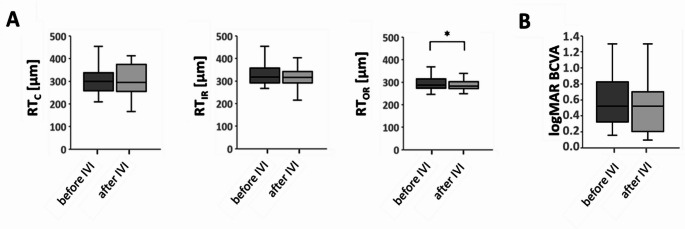
(**A**) Changes of RT in the fovea (**C**), the inner ring (IR) and the outer ring (OR) after ranibizumab injection. (**B**) BCVA in logMAR. * *p* < 0.05

RT decrease after IVI was statistically significant in OR (C: *p* = 0.794, IR: *p* = 0.490, OR: *p* = 0.038, Fig. 2A). The difference of BCVA was not significant (*p* = 0.076).

### Correlation analysis

Due to considerable interindividual variability in baseline values of FLT and retinal thickness, the analysis then focused on changes (Δ) before and after IVI. For further analysis, correlations between ΔBCVA, Δτ_1_, Δτ_2_, Δτ_m_, and ΔRT were evaluated by Spearman correlation analysis for both spectral channels (Table 2).

**Table 2.**
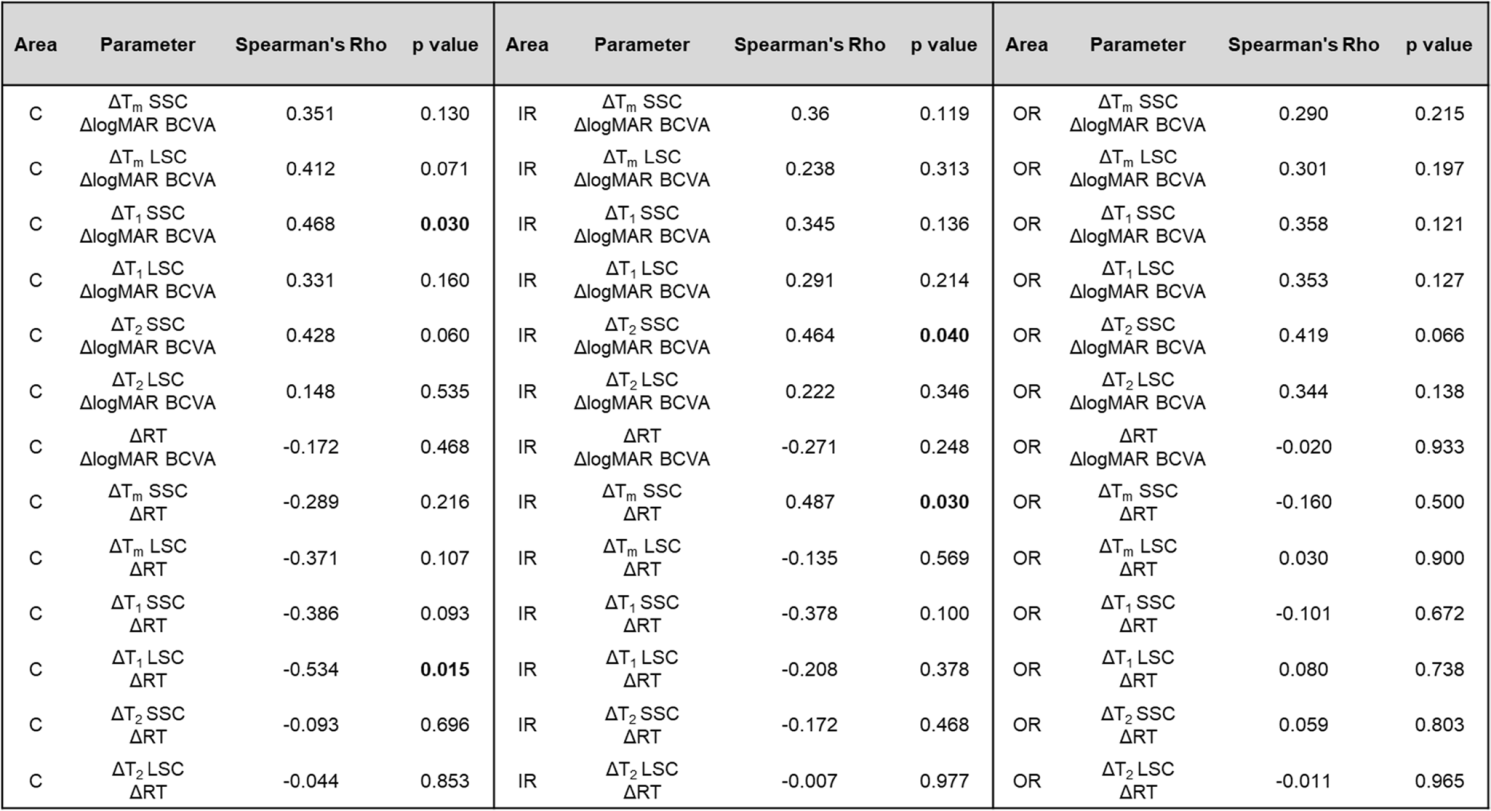
Spearman correlations between changes in BCVA, FLIO lifetime parameters (τ₁, τ₂, τm), and retinal thickness across retinal regions in both spectral channels

There were no significant correlations between ΔBCVA (logMAR) and ΔRT. In contrast, ΔBCVA (logMAR) showed a moderate and statistically significant correlation with Δτ_1_ in the central area of SSC (*ρ* = 0.468, *p* = 0.03) and with Δτ_2_ in IR of SSC (*ρ* = 0.464, *p* = 0.04). The positive correlations indicate that a greater improvement in visual acuity (i.e. a larger negative ΔBCVA in logMAR) was associated with a greater shortening of the FLT in SSC.

Furthermore, Δτ_1_ of the central area in the LSC and Δτ_m_ in the IR of the SSC correlated significantly with ΔRT. A decrease in retinal thickness was associated with a shortening of the FLT. However, there was no significant correlation between ΔBCVA and ΔRT.

These findings suggest that changes in FLT may reflect treatment-associated alterations that are related to both retinal structure and metabolic state.

**Fig. 3:**
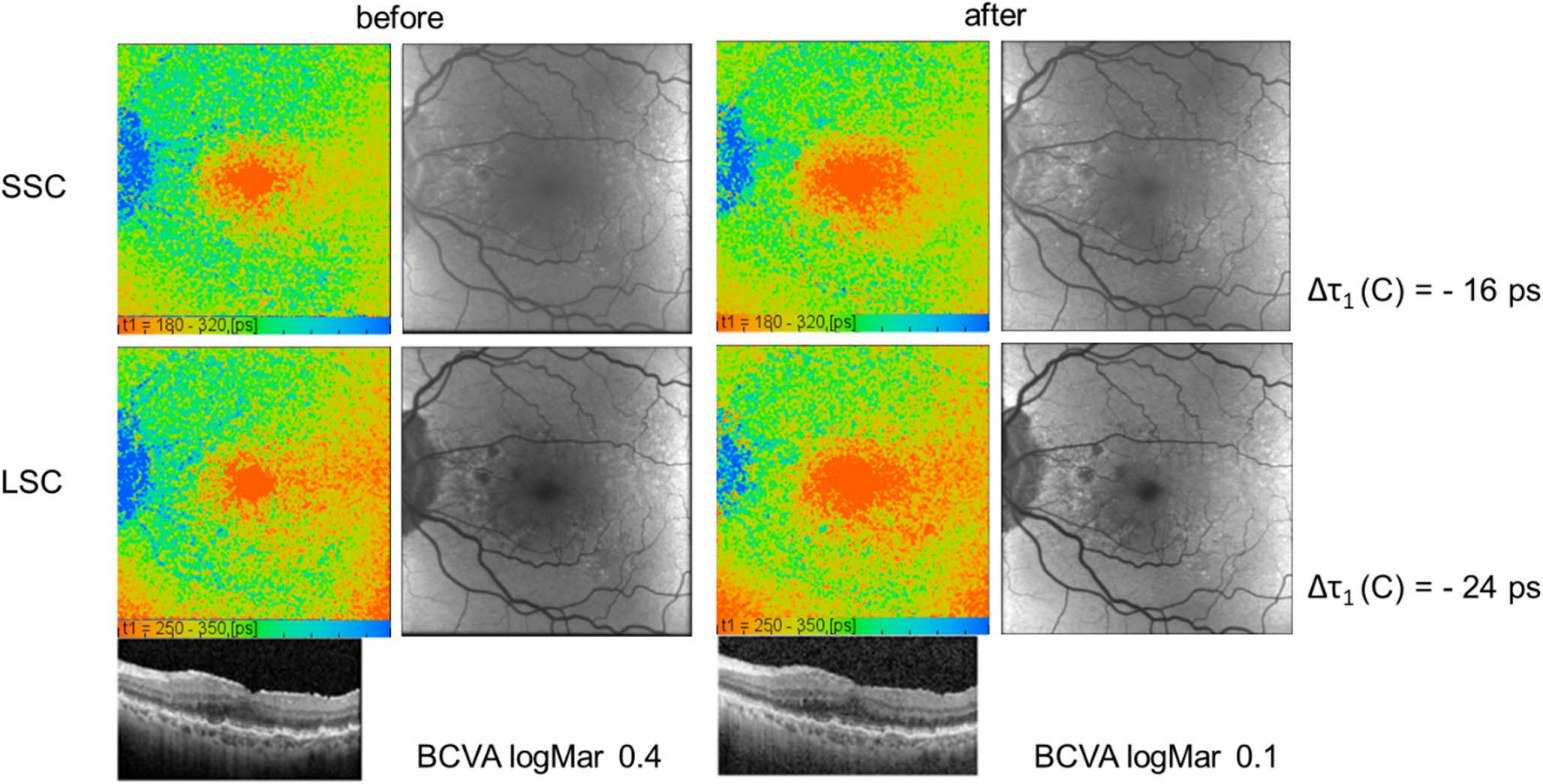
A representative case: Pseudo-colored image of τ_1_ (left) and autofluorescence intensity image (right) by FLIO, and Macula-OCT image of a 71-year-old man before and 4 weeks after ranibizumab IVI within the series of treatment (3/3). The shortening of τ_1_was seen in both spectral channels, along with an improvement in BCVA from logMar 0.4 to logMAR 0.1. Overall correlation of τ_1_ was statistically significant only in SSC

 Figure 3 shows an example case of one of the study patients, who showed an improvement of BCVA with shortening of τ_1_ in both channels but no change in RT. It should be noticed, that already before the IVI there was no longer intraretinal fluid. The IVI was nevertheless applied as it was the third out of a treatment series with 3 consecutive IVIs.

## Discussion

Neovascular AMD and its treatment with intravitreal anti-VEGF agents represent a significant financial burden for healthcare systems worldwide [17, 36]. Beyond the economic impact, patients often experience psychological stress due to frequent treatments, which may lead to therapy fatigue and reduced compliance [18, 37–39]. While these challenges may be considered acceptable if treatment leads to improvement, outcomes remain variable. A major concern in clinical practice is the group of non-responders, as the underlying reasons for differential treatment response are still not fully understood [16]. One potential explanation is the limitation of current diagnostic tools, which primarily reflect structural and functional changes, but may not fully capture the metabolic state of retinal tissue. The aim of this study was to evaluate whether fluorescence lifetime imaging ophthalmoscopy (FLIO) could provide additional metabolic insights during intravitreal anti-VEGF (ranibizumab) therapy in patients with nAMD.

Previous studies have shown that FLIO can detect early changes in AMD, even in the absence of apparent structural changes, which are thought to be related to alterations in retinal metabolism [26]. Our earlier FLIO investigations in AMD mouse models (ApoE^-/-^ and Nrf2^-/-^) also demonstrated FLT changes suggestive of altered retinal metabolism prior to morphological damage [40].

In the present study, we observed a reduction in retinal thickness (RT) (significant only in the outer ring) and an improvement in visual acuity, though not statistically significant. Interestingly, FLIO parameters (τ_1_, τ_2_, τ_m_) showed no significant change before and after treatment across all regions and channels. However, the absence of significant group-level changes likely reflects substantial inter-individual variability in FLIO responses. This heterogeneity prompted us to further explore associations between changes in FLIO parameters and functional outcomes using correlation analyses.

While a decrease in RT and improvement in vision are well-documented effects of anti-VEGF therapy [41], we found no correlation between RT and BCVA, which may reflect the heterogeneity of our cohort and the varying time points of measurement (e.g., before/after first vs. third injection). Notably, in some patients, RT remained stable due to absent intraretinal fluid, yet vision improved—possibly alongside metabolic changes detectable by FLIO.

This suggests that even after the third injection, functional and metabolic improvements may occur despite unchanged structural OCT findings. Our results align with the Inhibition of VEGF in Age-Related Choroidal Neovascularisation (IVAN) trial, which supported continued treatment, showing improved outcomes with sustained therapy [42].

Looking deeper into FLIO parameters, we found a significant positive correlation between changes in BCVA (ΔlogMAR) with Δτ_1_ in the central area of the SSC and with Δτ_2_ in IR of the SSC. Since logMAR improves as the values decrease, this means better visual acuity was associated with shorter τ_1_ and τ_2_ values. This aligns with findings from our previous study comparing smokers and non-smokers, where FLTs were significantly longer in smokers in the SSC [24], a group known to have impaired cellular metabolism and increased AMD risk [43–45]. These similarities, independent of structural OCT changes, support the idea that τ_1_ and τ_2_ reflect retinal metabolic state.

In another study, we found a negative correlation between serum total antioxidant capacity (TAC) and τ_2_ in the SSC, suggesting that oxidative stress may influence FLIO parameters [46]. Together, these findings indicate that τ_1_ and τ_2_ may be more sensitive indicators of retinal metabolism than the commonly used mean lifetime (τ_m_).

Given the well-established role of mitochondrial dysfunction in AMD pathogenesis [47, 48], a metabolic contributor to FLT changes in the SSC may include flavin-based cofactors involved in mitochondrial metabolism, such as FAD and FMN. Among these flavin cofactors, FAD is a major mitochondrial cofactor with an emission maximum at 524 nm (excited at 470 nm), which falls within the SSC detection range [19, 49]. FAD exhibits variable FLT depending on its binding state: free FAD shows relatively long lifetimes (~ 2300 ± 700 ps), whereas protein-bound FAD displays shorter lifetimes (~ 130 ± 20 ps for monomeric form; ~40 ± 10 ps for dimeric form) [50].

More recently, flavin mononucleotide (FMN) has also been reported to exhibit distinct FLT depending on its binding state, and its fluorescence properties, together with its close association with mitochondrial function, suggest that its contribution to SSC signals may not be negligible [51].

Accordingly, the shortening of τ_2_ after anti-VEGF therapy may reflect changes in the binding state and/or redox balance of flavin-based cofactors such as FAD and FMN, potentially indicating altered oxidative phosphorylation and mitochondrial activation [52–54]. Such metabolic changes may, in turn, be associated with changes in retinal cell function.

This interpretation is also supported by a study from Andrade Romo et al., which reported a decrease in flavoprotein associated fluorescence in patients with diabetic macular edema receiving anti-VEGF therapy and improved visual acuity. Those results were also interpreted as improved redox balance [55].

Additionally, we observed negative correlations between changes in RT and in Δτ_1_ in the central LSC and Δτ_m_ in the inner ring SSC. This suggests that shorter FLT values may also be associated with reduced RT, potentially reflecting improved retinal tissue homeostasis.

With respect to the LSC, interpretation of these findings is more challenging [56]. In general, LSC signals are highly influenced by lipofuscin, whose accumulation is known to lead to the longer lifetimes. However, substantial changes in lipofuscin accumulation are unlikely to occur over the short observation period of 4 weeks. Therefore, although lipofuscin-related effects cannot be entirely excluded, the observed FLT changes in the LSC should be interpreted with caution.

Interpretation of the FLIO results is limited by the fact, that FLIO values reflect the FLT values of all fluorophores at each pixel and the findings in our study can’t be attributed directly to a specific fluorophore. However, we wanted to highlight plausible biological mechanisms in our discussion and we assume, that the notable correlations in SSC are rather due to fluorophores, whose fluorescence properties can change dynamically with cellular metabolic state, than to relatively stable structural fluorophores.

Nevertheless, we cannot fully exclude that subtle microstructural changes, such as improved photoreceptor integrity (e.g., at the level of the ellipsoid zone), which may influence fluorescence lifetime, potentially through changes in light scattering. However, this mechanism is unlikely to be dominant in SSC, as similar lifetime shortening could be also observed in peripheral retinal regions outside the macular area, where macular pigments and pronounced photoreceptor structural changes play a lesser role.

Besides the difficulties in interpretation of FLIO results, the study is limited by its small sample size and the heterogeneity of the study population, including variation in disease duration and timing of imaging relative to injections. Subgroup analysis was not meaningful as the resulting small subgroups would lack sufficient statistical power and could be misleading.

These limitations suggest that larger and more homogeneous cohorts could provide more robust and reliable data, particularly for correlation analyses between changes in FLT and BCVA. Furthermore, BCVA assessments are subjective, although every effort was made to rule out confounding factors such as dry eye or other ocular conditions.

Due to the heterogeneous study group we also refrained from analyzing the integrity of the ellipsoidal zone as marker of retinal function even though this would be of high interest. The cohort includes patient with later states in which reliable segmentation of the ellipsoidal zone was not possible.

Larger, ideally prospective studies in treatment-naïve patients, as well as targeted investigations in non-responders, are needed to validate these preliminary findings and explore the clinical utility of FLIO in therapeutic decision-making.

In summary, this study provides evidence that FLIO may detect subtle metabolic changes in the retina following anti-VEGF therapy in patients with neovascular AMD. While structural imaging (OCT) remains the standard for monitoring, FLIO offers a complementary, non-invasive approach to assess retinal metabolic function, particularly through τ_1_ and τ_2_ parameters. These may serve as potential biomarkers for treatment response, helping to identify responders early and guide personalized therapy strategies. Future larger-scale clinical trials are warranted to further establish the role of FLIO in AMD management and treatment monitoring.

## Data Availability

No datasets were generated or analysed during the current study.
